# Social network changes during space restriction in zoo chimpanzees

**DOI:** 10.1007/s10329-018-0675-6

**Published:** 2018-07-17

**Authors:** Nicola F. Koyama, Filippo Aureli

**Affiliations:** 1 Research Centre in Evolutionary Anthropology and Palaeoecology, Liverpool John Moores University, Liverpool, UK; 2 Instituto de Neuroetologia, Universidad Veracruzana, Xalapa, Mexico

**Keywords:** Grooming, Aggression, Coping model, Crowding

## Abstract

Several studies across anthropoid species have demonstrated how primates respond to the increased risk of conflict during space restriction with various behavioral strategies. Three strategies have been proposed relating to tension regulation, conflict avoidance, and inhibition. Prior research supporting these strategies has focused on individual- and dyadic-level analyses, yet group-living animals live within a web of inter-individual connections. Here, for the first time, we used a network approach to investigate how social structure and individuals’ connectedness change during space restriction. We collected grooming and aggression data during a 6-week control period and a 5-week period of space restriction in a large group of zoo chimpanzees. We compared network density and individual centrality measures (degree, eigenvector, and betweenness centrality) between these two periods using permutation tests. The density of the unidirectional grooming network was significantly lower during space restriction, indicating fewer grooming partners and a less cohesive network. This was mainly due to a reduction in females’ grooming partners (degree) and an increase in females’ betweenness centrality. We found no differences in the mutual grooming or aggression networks. Our findings are consistent with a conflict avoidance strategy and complement previous findings from the same dataset based on individual behavioral rates that supported a selective inhibition strategy. The results highlight the dynamic nature of social structure and its inherent flexibility to respond effectively to short-term changes in the environment.

## Introduction

Much research has demonstrated how nonhuman primates flexibly use various coping strategies to avoid conflict and reduce social tension in response to reduced space availability (Anderson et al. 1977; Aureli et al. 1995; Aureli and de Waal 1997; Caperos et al. 2011; Caws and Aureli 2003; Cordoni and Palagi 2007; Crast et al. 2015; de Waal 1989; Duncan et al. 2013; Judge and de Waal 1993, 1997; Judge et al. 2006; Nieuwenhuijsen and de Waal 1982; Sannen et al. 2004; Tacconi and Palagi 2009; Videan and Fritz 2007; van Wolkenten et al. 2006). In contrast to an earlier influential study on rats that linked increased spatial density to increased aggression (The Density-Aggression Model, Calhoun 1962, validated in a range of species, e.g., dwarf mongoose *Helogale undulata rufula*, Rasa 1979; rabbits, *Oryctolagus cuniculus*, Myers 1966; baboons, *Papio anubis*, Elton and Anderson 1977; pigtailed macaques, *Macaca nemestrina*, Erwin and Erwin 1976), these studies have supported an alternative view: Under space restriction, social mechanisms are activated within groups of primates, such as avoiding potential aggressors and offering appeasement, which reduce the likelihood and/or intensity of aggression (The Coping Model, de Waal 1989). Different strategies may be used to cope with the possible negative consequences of reduced space availability depending on the circumstances. These strategies are based on the mechanisms primates use to manage conflict in a variety of contexts (Aureli and de Waal 2000).

One of these strategies is the tension-reduction strategy, where individuals increase affiliative and appeasement behaviors as spatial density increases in order to alleviate tension, increase tolerance, and minimize the likelihood of conflict escalation (Caperos et al. 2011; Crast et al. 2015; Duncan et al. 2013; Nieuwenhuijsen and de Waal 1982; Novak et al. 1992; Judge and de Waal 1997; Judge et al. 2006; Sannen et al. 2004; Videan and Fritz 2007). Another strategy is the conflict-avoidance strategy, where individuals reduce how often they actively seek interactions with others, leading to a decrease in affiliative behavior and a lack of aggressive escalation; whilst severe aggression does not increase, mild threats can increase (Aureli et al. 1995; Duncan et al. 2013; Judge and de Waal 1993; van Wolkenten et al. 2006; Videan and Fritz 2007). Thus, the outcome of a conflict-avoidance strategy would be little or no increase in overall aggression. A third strategy is the inhibition strategy, in which there is a reduction in aggressive interactions, in addition to a decrease in allogrooming and submissive behavior (Aureli and de Waal 1997).

A previous study by Caws and Aureli (2003) contributed to understanding the strategies primates use when space is reduced by focusing on the responses of a large group of zoo chimpanzees to the temporary reduction of escape opportunities. As the change in actual space use by the chimpanzees was less pronounced than in other studies focusing on the responses to reduced space availability, Caws and Aureli (2003) reported subtler behavioral changes. They did not find differences in overall aggression rates and allogrooming patterns between the period with a reduction of escape opportunities and the control period. However, during the period with reduced escape opportunities aggression rates decreased in dyads characterized by high aggression rates at baseline. These findings suggest that chimpanzees may adopt a selective inhibition strategy when escape opportunities are limited.

All studies reviewed above focused on behavioral changes at the individual or dyadic level. For example, behavioral rates of each individual were compared between conditions in Caws and Aureli’s study (2003) focusing on the effect of a temporary reduction of escape opportunities. Here we aimed to extend this approach by examining potential changes at the social structure level during the reduction in escape opportunities. Following Hinde’s (1976, 1979) framework, social structure is an emerging property based on the patterning of the social relationships among group members, and a social relationship is in turn based on the patterning of the different interactions exchanged between two individuals over time. Individuals can therefore be viewed as embedded in a network of inter-individual connections. Social network theory provides an array of centrality metrics that indicate an individual’s network position and thus how well the individual is integrated within its group (Croft et al. 2008; Sueur et al. 2011). These metrics, which are beyond the dyad level, give us insight into the individuals’ roles in network cohesion, and the factors that influence them, such as their sex (e.g., Flack et al. 2006; Kanngiesser et al. 2011). Recent studies have highlighted the importance of indirect connections in social cohesion, information transfer, cooperation and the adaptive value of social relationships (reviewed in Brent 2015).

Following this approach, we used social network analysis on allogrooming and aggression data collected for Caws and Aureli’s (2003) study to explore for the first time the impact of a reduction of space availability at the social structure level and examine whether the selective inhibition strategy chimpanzees used at the individual level held when their responses were analyzed at the social structure level. As mutual grooming has been found to reflect higher-quality relationships compared to uni-directional grooming in chimpanzees (Fedurek and Dunbar 2009), these two types of grooming may be affected differently by space restriction. Thus, we considered mutual grooming and unidirectional grooming separately. Within our data, a tension-reduction strategy would be recognized by an increase in grooming connectedness and no change in the aggression network; a conflict avoidance strategy would be supported by a decrease in affiliative connectedness and either no change or an increase in aggressive connections; whilst an inhibition strategy would involve a decrease in both affiliative and aggressive connections. When the number of social partners decreased, we were also interested in identifying which partners would be retained and which would not. Chimpanzees have sex-specific social strategies that are apparent both in the wild and captivity. Affiliation between males is higher than that between females, males are more aggressive than females and use more opportunistic social strategies during dominance competition (e.g., de Waal 1982; Goodall 1986; Nishida and Hosaka 1996). We therefore additionally analyzed male and female data separately. Specifically, we examined potential changes by comparing network positions (a) between periods for all adults, and males and females separately and (b) between males and females within periods.

## Methods

### Study subjects and site

The study group consisted of 29 chimpanzees located at Chester Zoo, United Kingdom. We conducted observations on the five sexually mature males (13–34 years) and 16 sexually mature females (8–53 years), excluding the eight immature individuals (seven females and one male; see Caws and Aureli 2003 for group history). The circular indoor enclosure (143-m^2^, 12-m high) contained a climbing frame and artificial termite mound, whilst the outdoor enclosure (2000-m^2^) was surrounded by a moat and contained a large grassy area and several tree trunks. In addition, there was a sleeping area of seven large interconnected pens that was not visible to the public. The chimpanzees usually had free access to the indoor and outdoor enclosure (but not the pens) during the day, and the indoor (but not the outdoor) enclosure and pens during the night. Water was freely available and the group was fed three times a day: in the indoor enclosure before the zoo opened and before observations began; a scatter feed in the outdoor enclosure in the afternoon around 14:30 h; and in the indoor enclosure after observations had terminated.

### Data collection

A trained research assistant collected data for 3 months from 19 January to 3 April 2000 between 10:30 and 16:30 but did not collect observations during the 14:30-h feeding time. The control period was from 19 January to 1 March. During this time, the chimpanzees had access to both the indoor and outdoor areas of their enclosure from around 9:00 h until around 17:00 h. They were fed in the outdoor enclosure in the afternoon however they stayed outdoors rarely due to the cold weather (e.g., the five sexually mature males were outdoors only about 15% of the time). The restricted period was from 2 March to 3 April, when the chimpanzees remained in the circular indoor enclosure because they could not access the outdoor enclosure, resulting in a reduction in escape opportunities. This situation was not completely novel to the chimpanzees, as they were occasionally restricted indoors during brief routine maintenance of the outdoor enclosure (e.g., about 3% of the time during the control period). Four females exhibited sexual swellings in both observation periods. Across both periods, up to six chimpanzees exhibited swellings daily.

**Fig. 1:**
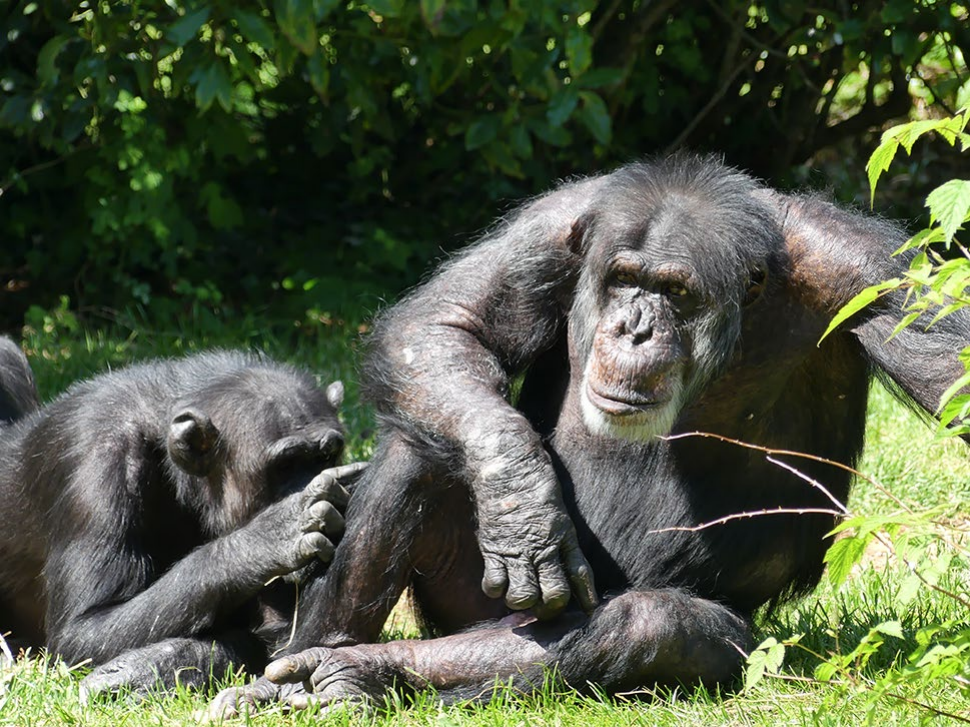
A female chimpanzee grooms a male chimpanzee

Allogrooming events were recorded via instantaneous sampling (Altmann 1974). Every 15 min, the whole group was scanned and the individuals involved in allogrooming were recorded, specifying whether it was a mutual grooming event (i.e., the two partners groomed each other simultaneously) or a uni-directional grooming event (i.e., one individual groomed the partner, Fig. 1). We carried out 433 scans (mean ± SD: 14.5 ± 2.5 per day) during the control period and 329 (15.6 ± 2.7 per day) during the restricted period. All instances of aggressive interactions (i.e., any behavior against another individual leading to screaming, or a bluff sequence leading to a submissive response or avoidance by another individual: van Hooff 1974) were recorded using all occurrence sampling (Altmann 1974) during 1-h observations 1–3 times a day. We are confident this sampling method was reliable because such aggressive interactions are conspicuous, being associated with loud vocalizations and occurred almost exclusively in a rather limited space: the indoor enclosure. We collected 51 h of all-occurrence observations during the control period and 52 h during the restricted period.

### Data analysis

We calculated network measures from non-valued data, using binary matrices of the presence/absence of behavior between individuals. Binary networks can be used to understand measures related to an individual’s number of social partners when the quality of observations is high (Croft et al. 2011). We considered all interactions, weak and strong, to be meaningful as we were not interested in interaction strength given the previous analyses at the individual level (Caws and Aureli 2003). We did, however, obtain results similar to Caws and Aureli (2003) when we ran the analysis using the weighted matrix (results not shown) i.e., there were no significant differences in any network metrics for unidirectional/mutual grooming or aggressive connectedness between the two periods. We constructed unidirectional grooming, mutual grooming and aggression networks for each period and calculated commonly used measures of centrality (Borgatti 2005) in UCINET 6.631 (Borgatti et al. 2002). We calculated binary degree (for behavior simultaneously exchanged) and binary indegree and outdegree (for directional behavior), to reflect the number of connections individuals maintained. We also calculated eigenvector centrality, which measures the extent to which an individual’s partners are connected to others in the network. Individuals with high eigenvector centrality have connections with partners who are themselves well connected. In addition, we calculated betweenness centrality, which reflects the number of shortest paths that pass through an individual linking other group members with each other. Individuals with high betweenness centrality are therefore more likely to have grooming partners who do not groom each another. We visualized network graphs using Netdraw in UCINET.

To analyze differences in the degree to which all individuals interacted we compared network densities (i.e., the proportions of all possible binary ties that were connected, which is a measure of network cohesion) between periods, using the *compare*-*densities* bootstrapping function in UCINET. We carried out paired permutation *t* tests using the *coin* package in R v. 3.4.1 (http://www.rproject.org) to compare all network metrics between periods. To further investigate the conflict avoidance strategy, where we found a difference in the number of partners that males and females groomed (outdegree) between periods, we examined tie strength to determine whether individuals preferentially maintained their strong ties rather than their weak ties. We used a paired permutation *t* test to compare the tie strength (percentage of scans spent grooming during the control period) of partners that were groomed in the restricted period (i.e., maintained) and those that were not (i.e., lost). In order to compare network metrics between males and females we calculated the probability of differences using node level permutation *t* tests in UCINET. We ran all tests using 10,000 permutations. All significant* p* values reported held under a sequential Bonferroni correction (Holm 1979) although we are aware such corrections are controversial and increase the likelihood of type II errors (e.g., Nakagawa 2004).

## Results

The mean (± SD) percentage of scans per individual spent in mutual grooming was 3.5 ± 3.6% in the control period and 4.5 ± 4.9% in the restricted period, and spent in unidirectional grooming given/received was 7.3 ± 3.6% in the control and 6.2 ± 3.4% in the restricted period. The mean hourly rate of aggression was 0.17 ± 0.30 in the control period and 0.14 ± 0.21 in the restricted period.

### Unidirectional grooming

**Fig. 2:**
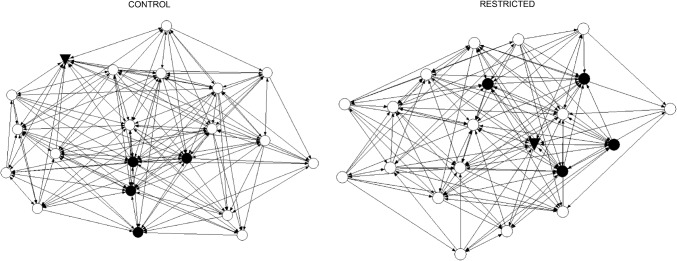
Chimpanzee social networks for unidirectional grooming during the control and restricted periods. Node color represents individual’s gender (males = *black circle*, females = *white circle*). The alpha male is indicated by the* black triangle* symbol. The spring-embedded layout places individuals with the smallest path lengths close to each other in the graph

We found that the density of unidirectional grooming ties was significantly lower in the restricted period (density = 0.37) compared to the control period (density = 0.49, *p* < 0.02, Fig. 2).

For unidirectional grooming by all adults, both the number of partners from whom the individual received grooming (indegree) and to whom the individual gave grooming (outdegree) were significantly lower in the restricted period (Table 1). This was due to females having fewer grooming partners in the restricted period, as for males there was no difference in the number of grooming partners between periods (Table 1). Four of the five males showed the same outdegree pattern as females did by grooming fewer partners during space restriction (7.8 ± 3.7) compared to the control period (11.5 ± 3.0), whereas the alpha male increased the number of partners he groomed from 5 to 10 partners during space restriction. Next, we checked whether females selectively reduced the number of partners that they groomed (outdegree), i.e., lost their weak ties but maintained their strong ties. The mean tie strength of partners that were not groomed in the restricted period (i.e., ‘lost’) was much weaker than for partners that were groomed in the restricted period (mean ± SD lost: 0.42 ± 1.1 and maintained: 1.1 ± 0.6, permutation *t* test: *z* = 2.86, *p* < 0.001).

**Table 1:** Permutation *t* test results comparing unidirectional grooming metrics between networks

	Centrality measure	*z*	*p*	Control (mean) ± SD	Restricted (mean) ± SD
Adults	Indegree	3.05	**0.003**	9.7 ± 3.4	7.4 ± 3.4
	Outdegree	2.64	**0.007**	9.7 ± 3.4	7.4 ± 2.2
	Eigenvector	0.14	0.89	0.21 ± 0.04	0.21 ± 0.05
	Betweenness	− 1.39	0.17	3.48 ± 2.47	4.24 ± 2.24
Females	Indegree	2.48	**0.013**	8.8 ± 3.2	6.6 ± 3.5
	Outdegree	2.47	**0.01**	9.6 ± 3.4	7.6 ± 1.8
	Eigenvector	0.22	0.83	0.20 ± 0.04	0.20 ± 0.05
	Betweenness	− 2.32	**0.011**	2.68 ± 2.1	3.84 ± 2.0
Males	Indegree	1.81	0.13	12.8 ± 2.3	8.2 ± 3.3
	Outdegree	1.02	0.37	10.2 ± 3.9	10.0 ± 1.4
	Eigenvector	− 0.11	0.88	0.25 ± 0.04	0.25 ± 0.02
	Betweenness	0.29	0.87	5.99 ± 1.84	5.52 ± 2.71

Significant differences are highlighted in bold

Betweenness centrality was higher for females in the restricted period than in the control period but there were no differences between periods for all adults or only males (Table 1). Eigenvector centrality did not differ between periods for all adults, only males and only females (Table 1). We did not find any differences between males’ and females’ unidirectional grooming centrality in the restricted period (all *p* > 0.05).

### Mutual grooming

We found no significant difference in the density of mutual grooming networks between the control and restricted periods, indicating that the proportion of possible ties that mutually groomed did not differ between periods (control = 0.36, restricted = 0.37, *p *= 0.69). Neither did we find any differences in network positions between periods for adults, females or males (all *p* > 0.05). There were no differences between male and female network measures in either the control or restricted period (all *p *> 0.05).

### Aggression

We did not find any difference in the proportion of ties that were aggressive between the control and restricted networks (control = 0.19, restricted = 0.19, *p* = 0.94). Neither did we find any differences in individuals’ network positions between the control and the restricted periods (all *p* > 0.05).

Males’ outdegree, eigenvector, and betweenness centrality in the aggression networks were significantly higher than females in both the control and restricted periods (Table 2). Males’ indegree was significantly lower than females’ indegree during the control although this was not the case in the restricted period (Table 2).

**Table 2:** Permutation *t* test results comparing male and female aggression metrics within networks

Period	Males (mean ± SD)	Females (mean ± SD)	*p*
Control			
Indegree	2.2 ± 0.98	4.38 ± 1.65	**0.017**
Outdegree	9.8 ± 4.12	2.0 ± 1.91	**0.0003**
Eigenvector	0.32 ± 0.16	0.12 ± 0.10	**0.007**
Betweenness	16.34 ± 15.20	2.69 ± 2.11	**0.004**
Restricted			
Indegree	3.6 ± 1.02	3.88 ± 2.18	0.81
Outdegree	9.8 ± 2.86	1.94 ± 1.92	**0.0004**
Eigenvector	0.39 ± 0.18	0.05 ± 0.04	**0.0001**
Betweenness	16.24 ± 13.13	4.80 ± 8.02	**0.02**

Significant differences are highlighted in bold

## Discussion

Little is known about whether or how individuals modify their social networks in response to changes in the physical environment. This is the first study to investigate the effect of reduced space availability on primate social networks. We investigated potential changes in social connectedness in zoo chimpanzees by comparing network positions during space restriction, creating a reduction in potential escape opportunities, with a control period. In a previous analysis of these data at the individual level, Caws and Aureli (2003) found only subtle changes in aggression rates in selected dyads and no changes in affiliation rates. It is perhaps not surprising then that we found that network density and individual centrality measures in the aggression networks were similar between periods. However, our network analysis detected changes in affiliation that were not apparent in the earlier study.

In contrast to Caws and Aureli’s (2003) findings of no change in individual grooming rates, here we found that the density of the network for unidirectional grooming was significantly lower during space restriction. Individuals groomed one another in almost half of all possible dyads in the control period, but only in over a third of dyads in the restricted period. The decrease in the overall density of the unidirectional grooming network was due to changes in network positions for females but not males: females reduced the number of partners that they groomed during space restriction by focusing on their stronger ties, that is, their core partners. This decrease in the affiliative network connectedness with no change in the aggression network is consistent with a conflict-avoidance strategy (Aureli et al. 1995; Judge and de Waal 1993) where individuals reduce active seeking of interactions with other group members. For example, increased huddling and reduced grooming in rhesus macaques (*Macaca mulatta*) under space-restriction conditions was interpreted as individuals ‘laying low’ amidst the security of their preferred partners (Judge and de Waal 1993). In addition, we found that betweenness centrality increased for females during space restriction, indicating that females were more likely to have grooming partners who did not groom each another, and that the overall network was less cohesive at this time. Network positions in the mutual grooming network were similar between periods, perhaps because this behavior is less sensitive to changes in social tension. Prior research suggests that mutual grooming functions as an efficient bonding mechanism in chimpanzees (Fedurek and Dunbar 2009). Therefore, it is possible that in our study partners who engaged in mutual grooming were those with higher-quality relationships that were resilient to the effects of potential increased social tension due to space restriction.

Overall, space restriction impacted social structure resulting in a less connected and less cohesive network. How this relates to network stability is not known but it is expected that individuals with high betweenness centrality become more important in maintaining network cohesion (Kanngiesser et al. 2011). Thus, social groups may be particularly vulnerable to fragmentation during periods of space restriction when social tension may be high. Space restriction often occurs in a captive environment to facilitate routine husbandry and can have important welfare consequences among primates (e.g., Ross et al. 2010; Pearson et al. 2015). Studies have begun to apply network analyses to detect social instabilities and manage social groupings to minimize stress (McCowan et al. 2008; Makagon et al. 2012; Rose and Croft 2015). In particular, network analyses can monitor group cohesion/fragmentation and consider social preferences and the identities of core social partners to inform management decisions about group composition and improve animal welfare.

Rather than reinforce the previous findings (Caws and Aureli 2003) of these same data indicating that individuals adopted a selective inhibition strategy, social network analysis uncovered an additional effect of space restriction on individual social behavior, which supports the adoption of a conflict avoidance strategy. This mixture of strategies mirrors previous findings about the use of various coping strategies during space restriction in different groups of chimpanzees. Aureli and de Waal (1997) reported an inhibition strategy in five groups, whereas Duncan et al. (2013) reported the use of a conflict-avoidance strategy in one group and a tension-reduction strategy in another group. Such evidence of a mixture of strategies within and between groups highlights the high degree of flexibility of chimpanzees’ behavioral responses to situations of potential increase of tension.

We found that females reduced the number of their grooming partners during space restriction and appeared to maintain more selective grooming networks by focusing on their stronger grooming ties. Our results are consistent with findings of baboon females (Wittig et al. 2008) and suggest the use of a conflict-avoidance strategy (Aureli et al. 1995; Judge and de Waal 1993). Females can potentially minimize their risk of aggression by reducing their level of movement and social activity at a time when enforced proximity and limited escape options increase the likelihood of conflict.

Chimpanzees are characterized by male philopatry and female dispersal, which impacts the nature of their social relationships: Males are more affiliative and aggressive than females and sex-specific roles are already present during infancy (e.g., Goodall 1986; Lonsdorf et al. 2014). Only one previous study has investigated how the effects of space availability on behavior are influenced by sex in chimpanzees. Videan and Fritz (2007) reported that males increased overall affiliation while decreasing aggressive behavior during short- and long-term space restriction, supporting the use of a tension-reduction strategy. Females did the same in the long term, but decreased both affiliative and aggressive behavior in the short term. Although the authors interpreted this as evidence for the conflict avoidance strategy, it would appear to provide support for the inhibition strategy. It should be noted that the overall tension-reduction strategy employed by males was largely due to males from one bachelor group, and the inhibition strategy shown by females was largely due to females in groups without adult males. It is difficult therefore to compare our findings with Videan and Fritz’s (2007) results but together they nonetheless highlight the behavioral flexibility of individuals to employ various strategies when risk of aggression is elevated during conditions of potential high tension.

We found that sex differences in network position within each period were evident only in the aggression network. As expected from previous studies (e.g., Goodall 1986; Muller 2002; Schel et al. 2013) males were more aggressive than females and occupied more central positions in both the control and restricted period: males’ outdegree, betweenness and eigenvector centrality were all higher than females. Thus, regardless of period, males were more likely to be aggressors and involved in aggression than females and were also more likely to be connected to other more aggressive individuals than females. Males’ indegree was lower than females’ indegree in the control period but not restricted period, indicating that males were less likely to be recipients of aggression in the control period compared to females.

Despite the sex differences noted above in wild chimpanzees, suggesting that males should occupy more central positions within their affiliative network, we did not find any differences in the grooming network positions between males and females. However, previous studies have noted that sex differences are influenced by captivity. In zoo chimpanzees, both males and females establish stable, high quality relationships (de Waal 1984, 1994; Fraser et al. 2008; Koski et al. 2012) which may account for the lack of difference in grooming network positions in our study.

Although previous studies investigating the effects of space restriction had much fewer adult males in their study groups, our analyses should be interpreted with caution because of the relative small sample size for adult males. We did, however, avoid manipulating group composition or introducing unfamiliar environments into our study design and so the fundamental difference between our two periods was the availability of space and escape routes. Nonetheless, our study is limited by the lack of an additional control period after the space restriction period. Thus we cannot rule out the possibility of order effects or changes over time in our results.

Although previous research provided support for various short- and long-term strategies during increased social tension, it is not clear at which point individuals may switch from a short-term conflict-avoidance mechanism to a long-term tension-reduction mechanism. Our period of space restriction lasted one month which falls in between definitions of short-term (days) and long-term (months) periods (e.g. Aureli and de Waal 1997; Videan and Fritz 2007). Rather than seeking evidence of discrete strategies adopted by groups, it may be more fruitful to examine how individuals flexibly adopt specific strategies according to their role in the group. For example, based on our individual data, whilst four of the adult males groomed fewer partners during the restricted period, the alpha male groomed twice the number of partners. Despite its rarity, evidence suggests that policing in chimpanzees is more likely during situations of social instability (von Rohr et al. 2012) when high ranking males, with sufficient social power to control instability, are more likely to intervene in conflicts. Considering the potential variation in individuals’ roles within the group it is likely that different coping strategies are employed during increased tension. In our study, it is possible that the alpha male sought to increase his social power within the group by increasing the number of partners groomed, at a time when other group members were reducing their connections.

Our findings extend prior research in two ways. First, by applying social network analysis to an area of research that typically uses individual or dyadic behavioral rates, we demonstrated its utility in increasing understanding of animals’ behavioral strategies. Indeed, by examining the group as a network, we found a reduction in the density of connections and the number of grooming partners during space restriction, patterns that were missed by the previous analysis at the individual level (Caws and Aureli 2003). Secondly, our results highlight sex differences in behavioral strategies to cope with space restriction, as we found that male chimpanzees employed an inhibition strategy, whilst female chimpanzees employed a conflict-avoidance strategy. Our network approach reveals the dynamic nature of social structure and its inherent flexibility to respond effectively to short-term changes in the environment.
